# A Population-Based Study of Four Genes Associated with Heroin Addiction in Han Chinese

**DOI:** 10.1371/journal.pone.0163668

**Published:** 2016-09-27

**Authors:** Yunxiao Li, Xiaomeng Qiao, Fangyuan Yin, Hao Guo, Xin Huang, Jianghua Lai, Shuguang Wei

**Affiliations:** 1 College of Forensic Science, Xi’an Jiaotong University, Key Laboratory of Ministry of Public Health for Forensic Science, Xi’an, PR China; 2 Key Laboratory of Environment and Genes Related to Diseases, Xi’an Jiaotong University, Ministry of Education, Xi’an, PR China; Kunming Institute of Zoology, Chinese Academy of Sciences, CHINA

## Abstract

Recent studies have shown that variants in FAT atypical cadherin 3 (*FAT3*), kinectin 1 (*KTN1*), discs large homolog2 (*DLG2*) and deleted in colorectal cancer (*DCC*) genes influence the structure of the human mesolimbic reward system. We conducted a systematic analysis of the potential functional single nucleotide polymorphisms (SNPs) in these genes associated with heroin addiction. We scanned the functional regions of these genes and identified 20 SNPs for genotyping by using the SNaPshot method. A total of 1080 samples, comprising 523 cases and 557 controls, were analyzed. We observed that *DCC* rs16956878, rs12607853, and rs2292043 were associated with heroin addiction. The T alleles of rs16956878 (*p* = 0.0004) and rs12607853 (*p* = 0.002) were significantly enriched in the case group compared with the controls. A lower incidence of the C allele of rs2292043 (*p* = 0.002) was observed in the case group. In block 2 of DCC (rs2292043-rs12607853-rs16956878), the frequency of the T-T-T haplotype was significantly higher in the case group than in the control group (*p* = 0.024), and fewer C-C-C haplotypes (*p* = 0.006) were detected in the case group. *DCC* may be an important candidate gene in heroin addiction, and rs16956878, rs12607853, and rs2292043 may be risk factors, thereby providing a basis for further genetic and biological research.

## Introduction

Heroin addiction is a chronic brain disease characterized by compulsive drug-seeking, drug abuse, physical dependence, tolerance, and relapse [1]. Heroin is one of the most commonly used drugs in China. At the end of 2014, a total of 2.955 million drug addicts were registered in China. Opioid drug addicts numbered 1.458 million, 49.3% of whom were heroin users. A total of 9.3 tons of heroin were seized in 2014. The direct economic losses resulting from drug addiction approach CNY 500 billion every year, representing a substantial economic burden to individuals and families. Similarly to other neuropsychiatric diseases, drug addiction results from a combination of genetic and environmental factors [2]. Family, adoption, and twin studies have suggested that genetic factors account for 30–60% of the overall variance in the risk of developing drug addiction [3–5].

Recently, FAT atypical cadherin 3 (*FAT3*), kinectin 1 (*KTN1*), discs large homolog 2 (*DLG2*) and deleted in colorectal cancer (*DCC*) have been reported to be associated with the function of the human mesolimbic reward system [6], which is the neurobiological basis of drug addiction. Cadherin, encoded by the *FAT3* gene, regulates neuronal morphology by affecting cell interactions [7], a crucial mechanism of pathological memory formation during drug addiction [8, 9]. The *FAT3* gene affects the volume of the caudoputamen [6], which plays important roles in habit formation, motivation, and the mechanism of drug addiction [10]. KTN1 is responsible for organelle transport and localization [11], and this protein is also closely associated with the formation and quantity of dendritic spines [12], which form the common anatomical substrate of drug addiction[9]. Another biological function of KTN1 is facilitating vesicle binding with kinesin, this binding is followed by kinesin-driven vesicle fast anterograde transport in axons [13, 14], suggesting that KTN1 is a promising candidate gene involved in drug addiction. Recently, the role of DLG2 has been investigated in a multitude of neuropsychiatric diseases. Genetic variants in *DLG2* affect learning and cognitive flexibility [15]. Genetic mapping of habitual substance users has revealed that DLG2 is overexpressed at the neural synapse [16]. The *DCC* gene encodes netrin-1 receptor, which affects axon guidance and migration [17]. *DCC* has been widely studied in a multitude of neuropsychiatric diseases. Sensitizing amphetamine pretreatment regimens result in selective upregulation of the expression of DCC in the ventral tegmental area of adult rodents [18], and *DCC* haploinsufficiency decreases sensitivity to the cocaine mediated enhancement of reward seeking behavior [19]. Furthermore, DCC is a regulator of maladaptive responses, such as tolerance, dependence and opioid-induced hyperalgesia to chronic morphine administration [20]. On the basis of these findings, these four genes may be important mediators of drug addiction. To the best of our knowledge, the roles of these genes in heroin addiction have not previously been reported.

Variations in gene functional regions may represent the most direct molecular mechanisms of disease[21]. The exon sequence can be transcribed into the final mRNA. Variations in exon regions may change the amino acid sequences. The most prominent example is brain-derived neurotrophic factor (*BDNF*), whose rs6265 SNP is directly associated with the clinical phenotype of drug addiction [22, 23]. Variations in promoter affect the efficiency of gene transcription. Variations in intron-exon borders may affect exon recognition and change the attributes of the alternative products [24, 25]. 5’UTRs are DNA regulatory sequences located in the 5’termini of protein-coding genes. These sequences can be transcribed to mRNA, but cannot be translated to protein. 5’UTRs contain a variety of regulatory elements, including the 5’cap, secondary structure, alternative 5’UTRs, internal ribosome entry sites, and upstream open reading frames (uORFs), among others. In general, 5’UTRs primarily regulate transcriptional initiation[26]. 3’UTRs are DNA regulatory sequences located downstream of the protein coding sequences, and these sequences primarily regulate gene expression at the post-transcriptional level, including transcriptional stability and cleavage, and polyadenylation, among others [27]. Because determining associations between functional polymorphisms and heroin addiction would be meaningful, we used HapMap (Han Chinese population) HCB data to systematically scan the promoter, 5’UTR, 3’UTR, exon, and intron-exon border regions of *FAT3*, *KTN1*, *DLG2* and *DCC*, and 20 SNPs were selected to do association analysis with heroin addiction.

## Materials and Methods

### Subjects

A total of 1080 individuals were recruited for the present study. All of these individuals were biologically unrelated individuals of China Han ancestry. Among them, 523 individuals were heroin addiction patients (mean age 45.13±7.270years) recruited from the Methadone Maintenance Treatment (MMT) Program at the Xi’an Mental Health Center between October 2013 and May 2015. At least two senior psychiatrists independently interviewed all patients, and urine testing and the Diagnostic and Statistical Manual of Mental Disorders, fourth revision (DSM-IVR) diagnostic criteria were applied to diagnose opioid addiction. A case vignette was generated to assist with the diagnosis, using a semi-structured interview with questions regarding (a) the age of onset and the duration of heroin use, (b) the quantity of the drug used during this period, (c) the route of administration (i.e., nasal inhalation or injection), (d) whether other substances were used or abused, and (e) comorbidity with any other psychiatric disorder. Participants meeting DSM-IVR criteria for an additional Axis I disorder; with a history of cigarette, alcohol, amphetamine, barbiturate, or benzodiazepine dependence; exhibiting mental illness or neurological diseases; or a history of hematological diseases, seizures, or other chronic physical illnesses were excluded.

The control cohort comprised 557 healthy people (mean age 45.80±10.449 years) recruited from the health examination center at the First Hospital Affiliated with the Medical College of Xi’an Jiao Tong University. The selection criteria were: having no individual history of drug addiction or mental illness, and frequency matching to cases on the basis of gender and age.

All participants provided written informed consent. Our study protocol was approved by the Ethical Committee of Xi’an Mental Health Center, Xi’an, China and the methods were performed in accordance with the approved guidelines.

### SNP selection

A total of 20 SNPs were selected on the basis of the following criteria: (1) location in functional region of the gene, including the promoter region, untranslated regions (UTRs), exons, and intron-exon borders, and (2) minor allele frequencies (MAF) greater than 0.05 on the basis of HapMap. The chromosomal positions of the six SNPs in *KTN1* (rs10146870, rs1138345, rs10483647, rs1951890, rs17128657, and rs945270) were searched from 55554095 to 55706484bp. The chromosomal positions of the six SNPs in *DCC* (rs17753970, rs934345, rs2229080, rs16956878, rs12607853, and rs2292043) were searched from 52338192 to 53536381bp. The chromosomal positions of the four SNPs in *FAT3* (rs10765565, rs4753069, rs2197678, and rs7927604) were searched from 92312328 to 92896960bp. The chromosomal positions of the four SNPs in DLG2 (rs575050, rs2512676, rs17145219, and rs2507850) were searched from 83454513 to 85629270bp. The databases were HapMap and dbSNP (HCB), and the positions of these SNPs are listed in Table 1.

**Table 1:** Genotypic and allelic frequencies of gene polymorphisms in the control and case group and statistical results.

Gene	Variable	Position	MAF	Controls (557)		Cases (523)		*P*-value^a^	*P*-value^b^	*P*-value^c^	OR, 95% CI
				No.	%	No.	%				
*FAT3*	rs10765565	Exon17	0.219					0.19	0.712	14.240	
	GG			345	61.9	321	61.4		0.849	16.980	0.976,0.764–1.248
	GT			180	32.3	177	33.8		0.594	11.880	1.071,0.831–1.381
	TT			32	5.7	25	4.8		0.478	9.560	0.824,0.481–1.410
	G allele			870	78.1	819	78.3		0.910	18.200	1.012,0.825–1.241
	T allele			244	21.9	227	21.7				
	rs4753069	Exon19	0.291					0.43	0.811	16.220	
	GG			284	51.0	269	51.4		0.883	17.660	1.018,0.802–1.293
	GA			222	39.9	201	38.4		0.632	12.640	0.942,0.738–1.203
	AA			51	9.2	53	10.1		0.586	11.720	1.119,0.747–1.676
	G allele			790	70.9	739	70.7		0.892	17.840	0.987,0.820–1.189
	A allele			324	29.1	307	29.3				
	rs2197678	3' UTR	0.289					0.178	0.432	8.640	
	CC			275	49.4	277	53.0		0.238	4.760	1.155,0.909–1.466
	CT			242	43.4	207	39.6		0.197	3.940	0.853,0.669–1.087
	TT			40	7.2	39	7.5		0.862	17.240	1.041,0.659–1.647
	C allele			792	71.1	761	72.8		0.392	7.840	1.086,0.900–1.310
	T allele			322	28.9	285	27.2				
	rs7927604	3' UTR	0.376					0.69	0.653	13.060	
	AA			219	39.3	201	38.4		0.765	15.300	0.963,0.754–1.231
	AG			257	46.1	254	48.6		0.425	8.500	1.102,0.868–1.400
	GG			81	14.5	68	13.0		0.463	9.260	0.878,0.621–1.243
	A allele			695	62.4	656	62.7		0.875	17.500	1.014,0.852–1.207
	G allele			419	37.6	390	37.3				
*KTN1*	rs10146870	5' near	0.403					0.33	0.562	11.240	
	GG			204	36.6	201	38.4		0.540	10.800	1.080,0.844–1.382
	GC			257	46.1	244	46.7		0.866	17.320	1.021,0.804–1.297
	CC			96	17.2	78	14.9		0.300	6.000	0.842,0.608–1.166
	G allele			665	59.7	646	61.8		0.326	6.520	1.090,0.917–1.296
	C allele			449	40.3	400	38.2				
	rs1138345	5' UTR	0.367					0.21	0.314	6.280	
	TT			230	41.3	229	43.8		0.407	8.140	1.107,0.870–1.410
	GT			245	44.0	233	44.6		0.852	17.040	1.023,0.805–1.301
	GG			82	14.7	61	11.7		0.138	2.760	0.765,0.536–1.091
	T allele			705	63.3	691	66.1		0.178	3.560	1.129,0.946–1.347
	G allele			409	36.7	355	33.9				
	rs10483647	Intron10	0.291					0.86	0.949	18.980	
	AA			281	50.4	269	51.4		0.746	14.920	1.040,0.819–1.321
	AG			228	40.9	210	40.2		0.794	15.880	0.968,0.759–1.235
	GG			48	8.6	44	8.4		0.904	18.080	0.974,0.635–1.494
	A allele			790	70.9	748	71.5		0.760	15.200	1.029,0.854–1.240
	G allele			324	29.1	298	28.5				
	rs1951890	Intron18	0.375					0.64	0.234	4.680	
	AA			220	39.5	218	41.7		0.465	9.300	1.095,0.859–1.396
	AG			256	46.0	247	47.2		0.677	13.540	1.055,0.828–1.337
	GG			81	14.5	58	11.1		0.090	1.800	0.733,0.511–1.051
	A allele			696	62.5	683	65.3		0.173	3.460	1.130,0.948–1.347
	G allele			418	37.5	363	34.7				
	rs17128657	Intron20	0.338					0.028	0.061	1.220	
	AA			256	46.0	249	48.2		0.587	11.740	1.069,0.841–1.357
	AT			226	40.6	222	42.9		0.532	10.640	1.080,0.848–1.376
	TT			75	13.5	46	8.9		0.015	0.300	0.620,0.420–0.914
	A allele			738	66.2	720	69.6		0.093	1.860	1.168,0.974–1.401
	T allele			376	33.8	314	30.4				
	rs945270	3' near	0.190					0.96	0.018	0.360	
	GG			365	65.5	310	59.3		0.034	0.680	0.766,0.598–0.980
	GC			172	30.9	177	33.8		0.298	5.960	1.145,0.887–1.478
	CC			20	3.6	36	6.9		0.015	0.300	1.985,1.134–3.475
	G allele			902	81.0	797	76.2		0.007	0.140	0.752,0.612–0.925
	C allele			212	19.0	249	23.8				
*DLG2*	rs575050	5' near	0.409					0.45	0.750	15.000	
	TT			190	34.1	186	35.6		0.616	12.320	1.066,0.830–1.370
	TG			278	49.9	249	47.6		0.450	9.000	0.912,0.718–1.158
	GG			89	16.0	88	16.8		0.707	14.140	1.064,0.771–1.468
	T allele			658	59.1	621	59.4		0.886	17.720	1.013,0.853–1.202
	G allele			456	40.9	425	40.6				
	rs2512676	3' UTR	0.317					0.69	0.146	2.920	
	TT			262	47.0	260	49.7		0.379	7.580	1.113,0.877–1.413
	TG			237	42.5	226	43.2		0.826	16.520	1.027,0.807–1.308
	GG			58	10.4	37	7.1		0.053	1.060	0.655,0.426–1.008
	T allele			761	68.3	746	71.3		0.128	2.560	1.153,0.960–1.387
	G allele			353	31.7	300	28.7				
	rs17145219	3’UTR	0.230					0.39	0.066	1.320	
	CC			334	60.0	338	64.6		0.114	2.280	1.220,0.953–1.561
	CG			190	34.1	168	32.1		0.488	9.760	0.914,0.709–1.178
	GG			33	5.9	17	3.3		0.037	0.740	0.533,0.293–0.970
	C allele			858	77.0	844	80.7		0.037	0.740	1.247,1.013–1.534
	G allele			256	23.0	202	19.3				
	rs2507850	3' near	0.311					0.52	0.146	2.920	
	GG			268	48.1	261	49.9		0.557	11.140	1.074,0.846–1.364
	GA			232	41.7	226	43.2		0.604	12.080	1.066,0.837–1.357
	AA			57	10.2	36	6.9		0.050	1.000	0.648,0.419–1.002
	G allele			768	68.9	748	71.5		0.192	3.840	1.131,0.940–1.360
	A allele			346	31.1	298	28.5				
*DCC*	rs17753970	5' near	0.499					0.19	0.311	6.220	
	AA			132	23.7	145	27.7		0.130	2.600	1.235,0.940–1.624
	AG			294	52.8	259	49.5		0.284	5.680	0.878,0.691–1.114
	GG			131	23.5	119	22.8		0.766	15.320	0.958,0.722–1.271
	A allele			558	50.1	549	52.5		0.266	5.320	1.101,0.930–1.303
	G allele			556	49.9	497	47.5				
	rs934345	5' near	0.286					0.17	0.021	0.420	
	GG			277	49.7	304	58.1		0.006	0.120	1.403,1.103–1.784
	GC			241	43.3	187	35.8		0.012	0.240	0.730,0.571–0.932
	CC			39	7.0	32	6.1		0.558	11.160	0.866,0.534–1.404
	G allele			795	71.4	795	76.0		0.014	0.280	1.271,1.048–1.541
	C allele			319	28.6	251	24.0				
	rs2229080	Exon3	0.497					0.77	0.195	3.900	
	CC			139	25.0	125	23.9		0.687	13.740	0.944,0.715–1.247
	GC			282	50.6	245	46.8		0.214	4.280	0.859,0.677–1.091
	GG			136	24.4	153	29.3		0.073	1.460	1.280,0.977–1.677
	C allele			560	50.3	495	47.3		0.171	3.420	1.125,0.950–1.332
	G allele			554	49.7	551	52.7				
	rs16956878	3' UTR	0.443					0.24	0.0001	0.002	
	CC			180	32.3	112	21.5		0.0001	0.002	0.571,0.434–0.751
	TC			261	46.9	260	50.0		0.348	6.960	1.121,0.883–1.424
	TT			116	20.8	148	28.5		0.004	0.080	1.500,1.135–1.984
	C allele			621	55.7	484	46.5		0.00002	0.0004	1.447,1.221–1.715
	T allele			493	44.3	556	53.5				
	rs12607853	3' UTR	0.443					0.20	0.0003	0.006	
	CC			180	32.3	113	21.7		0.0001	0.002	0.577,0.439–0.759
	CT			260	46.7	267	51.3		0.151	3.020	1.191,0.938–1.513
	TT			117	21.0	140	26.9		0.026	0.520	1.375,1.038–1.821
	C allele			620	55.7	493	47.4		0.0001	0.002	1.393,1.175–1.650
	T allele			494	44.3	547	52.6				
	rs2292043	3' UTR	0.425					0.16	0.001	0.020	
	TT			192	34.5	223	42.9		0.006	0.120	1.413,1.105–1.807
	TC			256	46.0	235	45.2		0.735	14.700	0.959,0.755–1.219
	CC			109	19.6	62	11.9		0.001	0.020	0.553,0.394–0.775
	T allele			640	57.5	681	65.5		0.0001	0.002	1.405,1.180–1.673
	C allele			474	42.5	359	34.5				

*P*-value ^a^ for Hardy-Weinberg equilibrium in controls.

*P*-value ^b^ for genotype and allele frequency difference.

*P*-value ^c^ for *P*-value ^b^ adjusted by Bonferroni correction.

### Genotyping

Peripheral blood samples from the enrolled subjects were collected in EDTA-coated tubes. Genomic DNA was extracted from blood leukocytes by using E.Z.N.A.^™^ Blood DNA Midi Kit (Omega Bio-Tek, Norcross, GA, USA) according to the manufacturer’s instructions. A total of 20 SNPs were genotyped by using SNaPshot SNP technology. A segment of DNA surrounding each SNP (151–368 bp) was amplified in a 10-μl PCR reaction containing 1XHotStarTaq buffer, 3.0 mM Mg^2+^, 0.3 mM dNTPs, 1 U of HotStarTaq polymerase (Qiagen Inc., USA), 1 μl of DNA and 1 μl of each PCR primer. The PCR program included an initial cycle at 95°C for 2 min, 11 cycles at 94°C for 20 s, 65°C for 40 s, and 72°C for 90 s, 24 cycles at 94°C for 20 s, 59°C for 30 s, and 72°C for 90 s, with a final cycle at 72°C for 2 min, and an indefinite hold at 4°C. To purify the PCR products, 5 U of shrimp alkaline phosphatase (SAP) enzyme and 2 U of Exonuclease I (Exo I) were mixed with 10 μl of the PCR product, incubated for 1 hour at 37°C and inactivated for 15 min at 75°C. The purified PCR products were used in a SNaPshot multiple single-base extension reaction. The extension reaction system (10 μl) contained 5 μl of the SNaPshot Multiplex Ready Reaction Mix (Applied Biosystems Co Ltd., CA, USA), 2 μl of the purified PCR product, 1 μl of the extension reaction primers, and 2 μl of ultrapure water. The PCR program initiated at 96°C for 1 min, and this was followed by 28 cycles of 96°C for 10 s, 55°C for 5 s, and 60°C for 30 s, and an indefinite hold 4°C. The products were purified after incubation with 1 U of SAP for 1 hour at 37°C, and this was followed by inactivation for 15 minutes at 75°C. Subsequently, 0.5 μl of the purified product was added to 0.5 μl of 120 Liz SIZE STANDARD (Applied Biosystems, Foster City, CA, USA) and 9 μl of Hi-Di (Applied Biosystems, Foster City, CA, USA), and this was followed by sequencing on an ABI3130XL Sequencer (Applied Biosystems, Foster City, CA, USA) after degeneration at 95°C for 5 minutes. The primary data were analyzed using GeneMapper 4.1 (AppliedBiosystems Co., Ltd., USA). The genotypes were determined on the basis of the nucleotide present at the SNP site, visualized as either one or two color peaks.

For quality control, 5% of the subjects (54 subjects) were randomly selected and blinded researchers conducted genotyping again, with a reproducibility of 100%.

### Expression quantitive trait locus analysis

The mRNA expression level and genotype data for significant SNPs were received from the SNPexp database (http://tinyurl.com/snpexp)[28]. The HapMap version for the genotype was HapMap2r23 unfiltered 3.96 million SNPs. The data form RNA expression levels were obtained from transcripts of lymphoblastoid cell lines from the same 45 unrelated Han Chinese individuals in Beijing. The correlations between the genotype and mRNA expression levels of significant SNPs were calculated by using linear regression and the Wald test.

### Statistical analysis

The genotype and allele frequencies of each individual polymorphism and the Hardy-Weinberg equilibrium (HWE) of the control and case groups were calculated by using the chi-square test. The associations between polymorphisms or other categorical variables with heroin addiction were assessed by using Pearson's Chi-square test. Continuous variables, such as the dose of heroin used and the age of heroin addiction onset, were analyzed using a correlate test. *P* values were calculated on the basis of the codominance or dominance of the rare allele, or the heterosis and recessive models of rare allele inheritance.

We computed pairwise LD statistics (D’ and r^2^) and haplotype frequencies using Haploview 4.0 (Broad Institute of MIT and Harvard, Cambridge, MA). We constructed haplotype blocks based on the criteria of Gabriel et al [29]. When the frequency of the haplotype was less than 5%, this value was excluded from the statistic analysis. We used PHASE 2.1.1 [30] software to verify the composition and frequency of positive haplotypes and to conduct permutation tests.

We analyzed the gene-gene interaction using Multifactor Dimensionality Reduction(http://sourceforge.net/projects/mdr/) which identifies high dimensional gene-gene interactions[31].

*P* values are presented as two-sided, and *p*<0.05 was considered statistically significant. We used Bonferroni’s correction to adjust the test level, and the *p* value was multiplied by all 20 loci or the haplotypes of each gene. All statistical analyses were conducted using SPSS 20.0 software (SPSS Inc., Chicago, IL, USA).

### Power analysis

A sufficient sample size was required in this genetics study [32]. Thus, we conducted a power analysis using a Power and Sample Size Program (http://biostat.mc.vanderbilt.edu/wiki/Main/PowerSampleSize).

## Results

**Fig 1:**
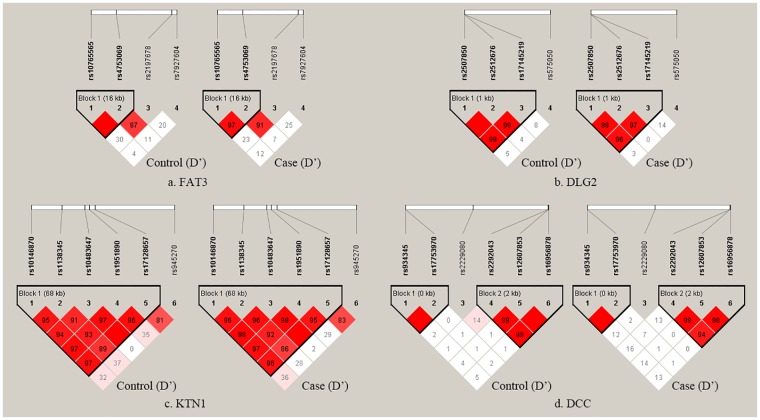
LD plot of the 20 SNPs in four genes in the control (left) and case (right) groups. (a) LD plot of the 4 SNPs of the *FAT3* gene in the control (left) and case (right) groups. (b) LD plot of the 4 SNPs of *DLG2* gene in the control (left) and case (right) groups. (c) LD plot of the 6 SNPs of the *KTN1* gene in the control (left) and case (right) groups. (d) LD plot of the 6 SNPs of the *DCC* gene in control (left) and case (right) groups. The values in squares are the D’ calculated using pair-wise analyses. Empty squares indicate D’ = 1 (i.e. complete LD between a pair of SNPs).

No significant deviation from HWE was observed for any of the SNPs in the case group. In the control group, the rs17128657 SNP statistically deviated from HWE (*p* = 0.028). Five blocks were identified in the linkage disequilibrium (LD) analysis of the case and control data. For the *KTN1* gene, block 1 contained five SNPs (rs17128657, rs1951890, rs10483647, rs1138345 and rs10146870). For the *DLG2* gene, block 1 contained three SNPs (rs2507850, rs2512676 and rs17145219). For the *FAT3* gene, block 1 contained two SNPs (rs10765565 and rs4753069). For the *DCC* gene, block 1 contained two SNPs (rs934345 and rs17753970), and block 2 contained three SNPs (rs2292043, rs12607853 and rs16956878) (Fig 1). The distributions, frequencies and statistical analyses of the genotype, allele, and haplotype are provided in Tables 1 and 2.

**Table 2:** The frequencies of haplotypes in the four genes and their associations with the risk of heroin addiction.

Gene	Block	Haplotype	Controls(557)		Cases(523)		*P*-value^a^	*P*-value^b^	OR, 95% CI
			No.	%	No.	%			
*KTN1*	block1	G-T-A-A-A	171	30.7	169	32.3	0.568	1.704	1.078,0.833–1.393
			386	69.3	354	67.7			
		C-G-A-G-T	169	30.3	139	26.6	0.171	0.513	0.831,0.638–1.083
			388	69.7	384	73.4			
		G-T-G-A-A	155	27.8	147	28.1	0.919	2.757	1.014,0.777–1.323
			402	72.2	376	71.9			
*DLG2*	block1	G-T-C	380	68.2	370	70.8	0.368	1.104	1.126,0.869–1.460
			177	31.8	153	29.3			
		A-G-G	127	22.8	98	18.7	0.100	0.300	0.781,0.581–1.049
			430	77.2	425	81.3			
		A-G-C	46	8.3	49	9.4	0.520	1.560	1.148,0.754–1.750
			511	91.7	474	90.6			
*FAT3*	block1	G-G	273	49.0	257	49.1	0.967	2.901	1.005,0.792–1.276
			284	51.0	266	50.9			
		G-A	162	29.1	153	29.3	0.951	2.853	1.008,0.775–1.311
			395	70.9	370	70.8			
		T-G	122	21.9	112	21.4	0.846	2.538	0.972,0.727–1.298
			435	78.1	411	78.6			
*DCC*	block1	G-A	279	50.1	275	52.6	0.413	1.239	1.105,0.870–1.403
			278	49.9	248	47.4			
		C-G	159	28.6	126	24.1	0.097	0.291	0.794,0.605–1.043
			398	71.5	397	75.9			
		G-G	119	21.4	123	23.5	0.396	1.188	1.132,0.850–1.507
			438	78.6	400	76.5			
*DCC*	block2	T-T-T	246	44.2	273	52.2	0.008	0.024	1.381,1.086–1.754
			311	55.8	250	47.8			
		C-C-C	237	42.6	175	33.5	0.002	0.006	0.679,0.530–0.870
			320	57.5	348	66.5			
		T-C-C	74	13.3	67	12.8	0.817	2.451	0.959,0.673–1.367
			483	86.7	456	87.2			

*P*-value^a^ based on comparison of frequency distribution of all haplotypes for the combination of SNPs

*P-*value^b^ for P-value^a^ adjusted by Bonferroni correction.

For the *DCC* gene, the rs16956878 and rs12607853 genotypes were strongly associated with heroin addiction (*p* = 0.002, and *p* = 0.006, respectively). The T allele frequencies of rs16956878 (*p* = 0.0004, odds ratio [OR] = 1.447, 95% confidence interval[CI] = 1.221–1.715) and rs12607853 (*p* = 0.002, OR = 1.393, 95%CI = 1.175–1.650) were significantly higher in the case group than in the control subjects. A significant difference was also observed in the distribution of the genotype frequency for rs2292043 between the case and control groups (*p* = 0.020). Compared with the control group, the case group exhibited a lower frequency of the C allele (*p* = 0.002, OR = 1.405, 95% CI = 1.180–1.673). For the KTN1 gene, addiction cases had a significantly higher frequency of the C allele than the control group at rs945270, but was not significant after Bonferroni correction (*p* = 0.140, OR = 0.752, 95%CI = 0.612–0.925). The sample size showed a 79%-91% power to detect associations with heroin addiction, with a presumed OR of 1.5, alpha value of 5%, and MAF ranging from 0.190 to 0.497.

In block 2 of *DCC* (rs2292043, rs12607853 and rs16956878), the frequency of the T-T-T haplotype was significantly higher than that in the control group (*p* = 0.024, OR = 1.381, 95% CI = 1.086–1.754), and fewer C-C-C haplotypes (*p* = 0.006, OR = 0.679, 95% CI = 0.530–0.870) were observed in the case group. The frequencies of these haplotypes in block 2 were similar to those obtained using PHASE (S1 Table). The *p* value was adjusted by using the 1000 permutations test (*p* = 0.026). On the basis of the results, we selected *DCC* rs16956878 as a representative of rs2292043, rs12607853 and rs16956878 for subsequent analyses.

We analyzed the mRNA expression level and genotype data for *DCC* rs16956878. The mRNA expression levels of subjects with TT and CC genotypes were similar, and showed no significant differences (S2 Table).

The demographic and addiction characteristics were analyzed with respect to *DCC* rs16956878 (Table 3). Compared with the CC genotype, the TT and TC genotypes were more likely to be associated with more varied routes of heroin administration. The TT and TC genotypes, compared with the CC genotype, were more likely to be associated with heroin use through sniffing, smoking, intravenous injection, or compound delivery methods.

**Table 3:** Demographic and addiction characteristics of *DCC* SNP rs16956878.

Variable	*DCC* rs16956878		
	CC	TC	TT
Age (year)	46.7±6.19	46.1±5.77	45.7±5.84
Gender (%)			
Male	21.3	49.8	28.9
Female	33.3	47.6	19.1
Occupation (%)			
Employed	19.2	54.1	26.7
Unemployed	23.1	47.4	29.5
Marital status (%)			
Unmarried	19.8	51.9	28.3
Married	23.0	48.0	29.0
Divorced or widowed	16.7	56.3	27.1
Route of heroin administration (%)^a^			
Sniffed or smoked	17.4	57.1	25.5
Injection via vein	21.9	51.7	26.5
Injection via muscle	36.4	36.4	27.3
Compound	26.5	27.9	45.6
Per-usage (gram)	0.3±0.14	0.3±0.34	0.3±0.18
Onset age (year)	29.1±6.68	29.4±6.41	28.6±6.73

^a^ Associated with route of heroin administration of rs16956878, p = 0.003.

The number that follows the ± sign is a standard deviation (s.d.).

The results of the gene-gene interaction using MDR are listed in Table 4. The testing balance accuracy and cross validation consistency were the highest in models of rs12607853, rs2229080, and rs934345 (S1 Fig). Because it had the highest cross validation consistency and testing balance accuracy, the three-locus model was considered to be the optimal model.

**Table 4:** The results of gene-gene interactions using MDR.

Model	Training Bal. Acc	Testing Bal. Acc	CV Consistency
rs16956878	0.5545	0.5526	8/10
rs16956878 rs934345	0.5802	0.5499	6/10
rs12607853 rs2229080 rs934345	0.6141	0.5824	10/10
rs12607853 rs2229080 rs2512676 rs934345	0.6618	0.5721	7/10

## Discussion

Addiction is a disease resulting from interactions between genes and the environment [33]. The genetic susceptibility of heroin addiction primarily refers to the likelihood of individuals to use or become addicted to heroin because of differences in genetic factors [34–36]. Family, adoption, and twin studies have suggested that genetic factors account for 30–60% of the overall variance in the risk of developing drug addiction [3, 4]. The aim of our research was to identify additional genetic markers of heroin addiction through a case-control study. In addition to genetics, the substances effect is also an important factor leading to heroin addiction [37]. Indeed, the effect is different among the same people receiving different doses of heroin in a certain range [38]. However, not all people exposed to heroin will become addicted to this drug[37]. Our heroin addicts were recruited from the MMT Program and were diagnosed with heroin addiction. Our healthy controls were never self-exposed to heroin. Thus, our results should indicate that some subjects are more likely to become addicted to heroin, despite the effects of environmental factors.

*DCC* is involved in axon guidance pathways, and its genetic variants influence the structure of the human mesolimbic reward system [6], which plays a key role in drug addiction. Our study provides direct evidence that polymorphisms of the *DCC* gene are associated with heroin addiction in the Chinese Han population.

In the present study, we observed that the T alleles of the *DCC* SNPs rs16956878 and rs12607853 were strongly associated with an increased risk of heroin addiction, whereas the C allele of *DCC* rs2292043 was associated with a decreased risk of heroin addiction, and these variants are located within the *DCC* 3’ UTR. Moreover, we observed a significant increase in the T-T-T haplotype (rs2292043- rs12607853- rs16956878) in heroin addicts compared with the members of the control group. These results suggest that the subjects carrying the T-T-T haplotype are more likely to become addicted to heroin. The 3’ UTR of a gene contains a number of regulatory sequences that are targets of a variety of regulatory molecules, including RNA binding proteins (RBPs) and small noncoding RNAs (ncRNAs), which recognize small cis-elements present in the 3’ UTRs and determine the stability, cellular localization, and translation of the encoded mRNA [39, 40]. Among these regulatory molecules, microRNAs down-regulate genes and promote RNA cleavage through perfect base pairing with a target sequence [27]. By searching the MirSNP database [41], we observed that when rs12607853 allele changes from T to C, the mRNA of *DCC* can combine with hsa-miR-141-3p, and decrease *DCC* gene expression. When the rs2292043 allele changes from T to C, the mRNA of *DCC* can combine with hsa-miR-141-3689d, and decrease *DCC* gene expression. When the rs16956878 allele changes from T to C, the combined effect of the mRNA of *DCC* and hsa-miR-4666a-5p increases, thereby decreases *DCC* gene expression. Thus, in the heroin group, the higher frequency of T than C alleles, led to increased RNA expression. Our results are in agreement with the previous animal experimental conclusions. *DCC* haploinsufficiency mice showed blunted sensitivity to cocaine-mediated enhancement of reward seeking behavior [19]. We conducted an eQTL analysis of the mRNA expression level and examined the genotype data for *DCC* rs16956878 obtained from the SNPexp database. The mRNA expression levels of the subjects with TT and CC genotypes showed no significant differences. Because the RNA expression level and genotype data were obtained from only 45 unrelated individuals, the results may reflect the small sample size. Therefore, additional studies on the mRNA expression level of *DCC* and examination of the genotype data for rs16956878 with larger sample sizes are urgently needed. Grant et al. have reported an association between schizophrenia and the rs2270954 polymorphism in the 3’ UTR of the *DCC* gene [42]. Peng et al have reported an association between schizophrenia and the rs2229080 polymorphism in the exon 3 of the *DCC* gene [43]. These results further support an important role for DCC in neuropsychiatric diseases. To confirm the link between the *DCC* gene and addiction, rs16956878 was analyzed, and the results suggested an association with the route of heroin administration. The rs16956878 TT and TC genotypes were associated with increased variance in the route of heroin administration and therefore might be associated with easier access to drugs. DCC is involved in axon guidance pathways and plays a critical functional role in the organization of brain development and in adult neuroplasticity [17, 44]. These results suggest that the *DCC* gene may contribute to the genetic basis of individual differences in susceptibility to heroin addiction.

*KTN1* encodes the protein kinectin, which is primarily found in the endoplasmic reticulum in the dendrites and thesoma of neurons [12]. KTN1 plays a critical role in the regulation of neuronal cell shape, spreading, and migration through kinectin–kinesin interactions [45]. Disrupting the kinectin–kinesin interaction results in a morphological change to a rounded cell shape and reduced cell spreading and migration[45], which decreases the polarization of the neuronal architecture and the cellular complexity essential for neuronal functions [46]. Therefore, KTN1 may affect the density or complexity of the dendritic spines in drug addicts, thereby causing brain region-specific changes in the density of these structures[47]. The rs945270 SNP is located 50 kb downstream of the *KTN1* gene of 14q22.3. The C allele of rs945270 increases the expression of *KTN1* in the frontal cortex and putamen [48, 49]. In the present study, we identified a significantly higher C allele frequency in the heroin addiction group, although this result was not significant after correction. It has been suggested that subjects with the C allele in addicts might exhibit higher *KTN1* expression in the frontal cortex and putamen. Interestingly, amphetamine, cocaine and nicotine increase the spine density on the apical dendrites of the medial prefrontal cortex [50–52] and morphine significantly increases dendritic spine density in the orbital frontal cortex of adult rats[47]. Thus, KTN1 may increase the density or complexity of dendritic spines in the frontal cortices of heroin addicts.

FAT3 is the human homolog of *Drosophila* FAT which inhibits Yokie through phosphorylation and subsequently activates the expanded-Hippo-Warts signaling cascade[53]. Phosphorylation of yes-associated protein 1(YAP1) in Hippo signaling inhibits the Wnt signaling cascade through interactions with β-catenin [54]. The cell polarity protein complex, Dlg/Lgl/Scrib affects the cell-cell contacts, thus leading to the deregulation of the actin cytoskeleton through interactions with Hippo pathways[55]. Netrin and Wnt signaling pathways play important roles in axon guidance[56]. Netrin signaling is primarily responsible for dorso-ventral (D/V)-graded distributions and Wnt signaling is primarily responsible for antero-posterior (A/P) distributions[57]. Kinesin-1 acts with DCC in sensory neuron position [58]. Thus, we speculated that these four genes might be involved in Hippo and/or Wnt signaling pathways. Studies have shown that the Wnt pathway regulates the susceptibility of chronic stress and addiction through the regulation of the differentiation of dopamine neurons in the mesolimbic reward system [59, 60]. Unfortunately, we did not obtain direct evidence from the KEGG pathway and PATHWAY STUDIO databases. The SNPs in the optimal model of gene-gene interaction were rs12607853, rs2229080, and rs934345, all of which are located in the *DCC* gene and no gene-gene interactions were detected. Thus, a pathway study of these genes would be meaningful in the future.

## Conclusion

To the best of our knowledge, this is the first report demonstrating an association between heroin addiction and functional polymorphisms within the *DCC* gene in a homogeneous sampling population. However, further replication or validation across populations should be considered in the future. Moreover, studies of these polymorphisms and their expression are warranted to further the understanding of how these variants influence the expression and induction of these genes. These studies should help to elucidate the pathogenesis of heroin addiction and may offer a basis for the diagnosis and treatment of addiction.

## Supporting Information

S1 Dataset Genetic data in this study. (XLSX)

S1 Fig Optimal models determined by using MDR. Graphical model of rs12607853, rs2229080, and rs934345 (for SNP: 0 = no risk alleles, 1 = 1 risk allele, and 2 = 2 risk alleles). In each small square, the numbers represent the number of cases (left) and controls (right). Dark-shading for each square represents a high risk of disease, whereas light shading indicates a low risk of disease.
(TIF)

S1 Table Haplotype frequencies estimated by using PHASE. (DOCX)

S2 Table *DCC* mRNA expression in the genotypes of rs16956878. (DOCX)
