# Supplementary figures and images for: A Population-Based Study of Four Genes Associated with Heroin Addiction in Han Chinese
Source: PLoS One. 2016 Sep 27;11(9):e0163668. doi: 10.1371/journal.pone.0163668 (PMC5038970; doi:10.1371/journal.pone.0163668)

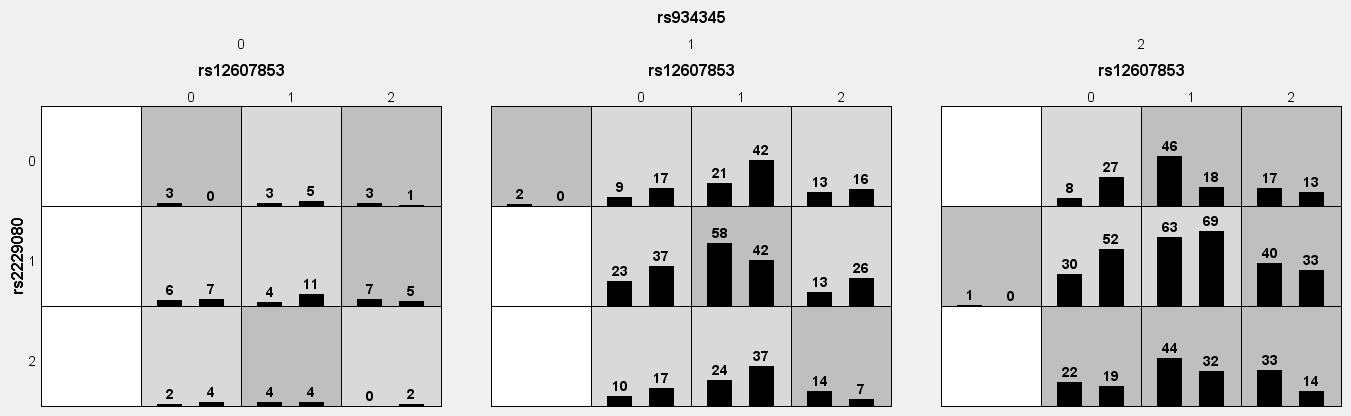

Supplement: S1 Fig — Graphical model of rs12607853, rs2229080, and rs934345 (for SNP: 0 = no risk alleles, 1 = 1 risk allele, and 2 = 2 risk alleles). In each small square, the numbers represent the number of cases (left) and controls (right). Dark-shading for each square represents a high risk of disease, whereas light shading indicates a low risk of disease. (TIF) [file pone.0163668.s002.tif]
