# Supplementary material for: A Population-Based Study of Four Genes Associated with Heroin Addiction in Han Chinese
Source: PLoS One. 2016 Sep 27;11(9):e0163668. doi: 10.1371/journal.pone.0163668 (PMC5038970; doi:10.1371/journal.pone.0163668)
Supplement: S1 Table — (DOCX) [file pone.0163668.s003.docx]

**S1 Table** Haplotype frequencies estimated by PHASE

| Variable | Total/% | Case/% | Control/% | ***P*-value**^a^ |
| --- | --- | --- | --- | --- |
| T-T-T | 48.04 | 52.18 | 44.15 | 0.026 |
| C-C-C | 38.11 | 33.49 | 42.44 |  |
| T-C-C | 13.05 | 12.87 | 13.21 |  |

***P*-value**^a^ is adjusted by permutation test, and the displacement is 1000 times.
