# Supplementary material for: A Population-Based Study of Four Genes Associated with Heroin Addiction in Han Chinese
Source: PLoS One. 2016 Sep 27;11(9):e0163668. doi: 10.1371/journal.pone.0163668 (PMC5038970; doi:10.1371/journal.pone.0163668)
Supplement: S2 Table — (DOCX) [file pone.0163668.s004.docx]

**S2 Table** DCC mRNA expression by the genotypes ofrs16956878

| CHR | SNP | VALUE | G11 | G12 | G22 | P |
| --- | --- | --- | --- | --- | --- | --- |
| 18 | rs16956878 | GENO | C/C | C/T | T/T | 0.1428 |
| 18 | rs16956878 | COUNTS | 9 | 26 | 10 |  |
| 18 | rs16956878 | FREQ | 0.2 | 0.5778 | 0.2222 |  |
| 18 | rs16956878 | MEAN | 5.961 | 5.886 | 5.951 |  |
| 18 | rs16956878 | SD | 0.03615 | 0.08338 | 0.09988 |  |
